# Deoxynivalenol and its toxicity

**DOI:** 10.2478/v10102-010-0019-x

**Published:** 2010-09

**Authors:** Pavlina Sobrova, Vojtech Adam, Anna Vasatkova, Miroslava Beklova, Ladislav Zeman, Rene Kizek

**Affiliations:** 1 Department of Chemistry and Biochemistry, Faculty of Agronomy, Mendel University in Brno, Zemedelska 1, CZ-613 00 Brno, Czech Republic; 2 Department of Animal Nutrition and Forage Production, Faculty of Agronomy, Mendel University in Brno, Zemedelska 1, CZ-613 00 Brno, Czech Republic; 3 Department of Veterinary Ecology and Environmental Protection, Faculty of Veterinary Hygiene and Ecology, University of Veterinary and Pharmaceutical Sciences, Palackeho 1-3, CZ-612 42 Brno, Czech Republic

**Keywords:** Deoxynivalenol (DON), *Fusarium*, grain, mycotoxin, toxicity

## Abstract

Deoxynivalenol (DON) is one of several mycotoxins produced by certain *Fusarium* species that frequently infect corn, wheat, oats, barley, rice, and other grains in the field or during storage. The exposure risk to human is directly through foods of plant origin (cereal grains) or indirectly through foods of animal origin (kidney, liver, milk, eggs). It has been detected in buckwheat, popcorn, sorgum, triticale, and other food products including flour, bread, breakfast cereals, noodles, infant foods, pancakes, malt and beer. DON affects animal and human health causing acute temporary nausea, vomiting, diarrhea, abdominal pain, headache, dizziness, and fever. This review briefly summarizes toxicities of this mycotoxin as well as effects on reproduction and their antagonistic and synergic actions.

## Introduction

*Fusarium* mycotoxins are the largest group of mycotoxins, which includes more than 140 known metabolites of fungi. They are synthesized by many species of fungi, mainly by *Fusarium* (*F. graminearum* and *F. culmorum*). Due to the high toxicity of *Fusarium* mycotoxins and high occurrence of the fungi species producing them, these mycotoxins belong to the most animal and human health endangering ones. They are abundant in cereals and their products (Yazar & Omurtag, 2008). Deoxynivalenol, nivalenol and T-2 toxin belong to the most occurred *Fusarium* mycotoxins.

## Trichothecenes and deoxynivalenol

Deoxynivalenol (DON) is a natural-occurring mycotoxin mainly produced by *Fusarium graminearum* (Kushiro, 2008). It is also know as vomitoxin due to his strong emetic effects after consumption, because it is transported into the brain, where it runs dopaminergic receptors. The emetic effects of this mycotoxin were firstly described in Japanese men consuming mouldy barley containing *Fusarium* fungi in 1972 (Ueno, 1985; Ueno, 1988). DON is probably the best known and most common contaminant of grains and their subsequent products. Its occurrence in food and feed represent more than 90% of the total number of samples and it is a potential marker of the occurrence of other mycotoxins.

**Figure 1:**
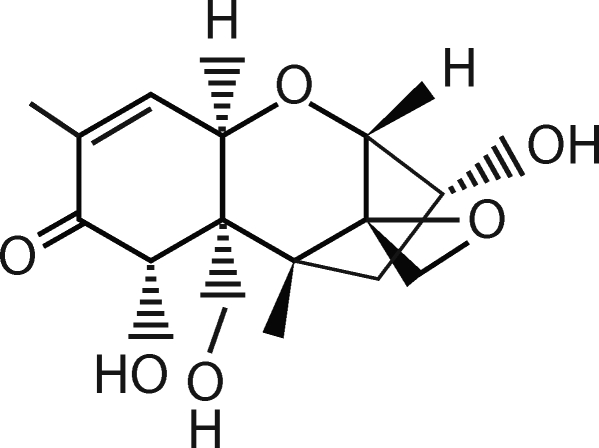
Chemical structure of deoxynivalenol (DON).

Chemically DON is a member of the trichothecenes family of mycotoxins (Figure 1). Structurally, it is a polar organic compound, which belong to the type B trichothecenes and its chemical name is 12,13-epoxy-3α,7α,15-trihydroxytrichothec-9-en-8-on (Nagy *et al*., 2005). In its molecule it contains 3 free hydroxy groups (-OH), which are associated with its toxicity. From its chemical structure its physical and chemical properties shown in Table 1 follow.

**Table 1:** Physico-chemical properties of deoxynivalenol.

Property	Information
Name	Deoxynivalenol (DON), vomitoxin
IUPAC name	12,13-epoxy-3α,7α,15-trihydroxytrichothec-9-en-8on
Molecular formula	H_15_O_20_O_6_
Molar mass	296.32 g/mol
Physical state	Colourless fine needles
Boiling Point (°C)	543.9 ± 50.0°C
Melting Point (°C)	151–153°C
Flash Point (°C)	206.9 ± 2.5
Vapour Pressure (Torr)	4.26×10^–14^ 25°C
Soluble in:	polar organic solvents (*e.g*., aqueous methanol, ethanol, chloroform, acetonitrile, and ethyl acetate) and water

One of the most important physicochemical property of DON is its ability to withstand high temperatures, which is the risk of its occurrence in food (Hughes *et al*., 1999). Numerous studies have documented that DON was heat-stable. DON is very stable under temperature within the interval from 170°C to 350°C, with no reduction of DON concentration after 30 min at 170°C. However, DON levels are reduced in cooked pasta and noodles because of leaching into the cooking water (Manthey *et al*., 2004; Sugita-Konishi *et al*., 2006; Visconti *et al*., 2004), because DON is water-soluble, but no reduction of its concentration was observed during frying DON-contaminated food in oil. Some evidence indicates that DON levels may be reduced during the processing, mainly boiling in water, Chinese noodles containing Kansui: a commercial preparation of potassium and sodium carbonate and phosphate salts (Kushiro, 2008).

### Deoxynivalenol in human and animal health

**Figure 2:**
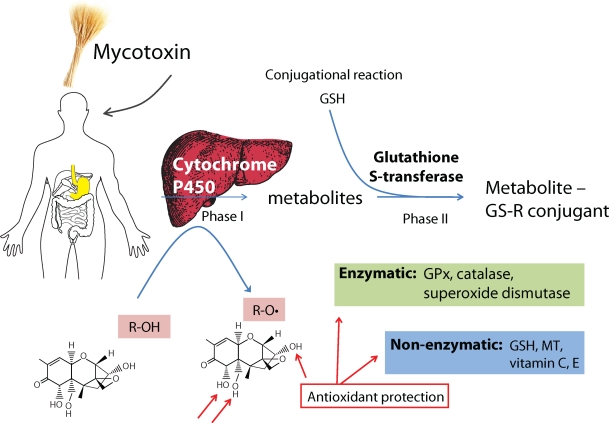
Scheme of the possible way of deoynivalenol detoxification. The first and one the most important pathways using for detoxifying of DON is cytochrome P450, which serves to catalyze the oxidation of organic substances. This pathway, however, can cause that free hydroxyl groups of DON can be cleaved and DON-radical can be more dangerous. The DON-radical can be scavenged by enzymatic (glutathione peroxidase (GPx), catalase, superoxide dismutase) or non-enzymatic (reduced glutathione (GSH), metallothionein (MT) and vitamins) ways. Nevertheless, cytochrome P450 can be followed phase II, in which glutathione-S-transferase can form conjugate with GSH and DON, which results in detoxification of the xenobiotic.

Potential impact of DON on human health may occur after ingestion of contaminated foods from oats, barley, wheat, corn or other grains. DON was detected also in buckwheat, sorghum, popcorn and other foods for human consumption, such as flour, bread, noodles, beer and malt (Pestka and Zhou, 2000). For more details also see “Deoxynivalenol. Safety Evaluation of Certain mycotoxins in food” (www.inchem.org). Danger resulting from this is that the toxin still remains in foods and feeds after basic culinary treatment. DON does not constitute a significant threat to public health. In a few cases short-term nausea and vomiting have been recorded (Perkowski *et al*., 1990). Other effects include diarrhea, abdominal pain, headache, dizziness and fever. Research on beer samples showed the presence of DON in fermented beers in Holland, which ranged from 26 to 41 mg/L [0.088 to 0.14 µM]. In the case of German beers this figure goes above 200 ng/ml [0.675 µM] (Schothorst and Jekel, 2003). Another beer samples obtained from the European chain stores showed values ranging from 4.0 to 56.7 ng/mL [0.013 and 0.191 µM] (Papadopoulou-Bouraoui *et al*., 2004). Scheme of potential detoxification of DON is shown in Figure 2.

In animals that were exposed to the DON it was found the subsequent transfer of this toxin to animal products. However, the rate of transmission was low. DON concentrations were monitored in the blood plasma, bile, liver, kidneys, thigh muscle and the dorsal fat in pigs which were feed over 11 weeks of 25 or 50% contaminated wheat with 2.5 mg/kg DON. The content of DON in muscle tissue was 2.2 ng/g in pigs fed by 25% contaminated wheat and 5.2 ng/g in pigs fed by 50% contaminated wheat. In the liver, the content was 3.6 ng/g for 25% experimental animals, and 4.8 ng/g for 50%. In the kidneys, this content was app. same for both experimental variants as 19.3 ng/g. In addition, metabolite de-epoxy-DON was detected only in liver (0–2.4 ng/g) (Doll *et al*., 2008). The maximum recalculating factor (the sum of the DON and de-epoxy-DON in tissues divided by concentration of DON administered in the diet) was 0.0043 for muscle 0.0064 for liver and 0.0319 in the kidney (Doll *et al*., 2008). These factors are very similar to those of other studies performed on pigs, which were fed with a diet contaminated by DON (6.68 mg/kg) for 12 weeks. The highest recalculating factors were 0.0031, 0.0059 and 0.0193 for muscle, liver and kidneys, respectively. DON was also detected in the dorsal fat in pigs (Goyarts *et al*., 2007). In spite of the consuming animal tissues exposed to the DON secondary intoxication is negligible.

Other animal products, which may be hazardous to humans, are eggs. Nevertheless, DON level in eggs compared to other raw materials of animal origin is negligible as it follows from the study with hens fed diets containing DON (~20 mg/kg [67 µmol/kg]) (Sypecka *et al*., 2004). The other concern is milk. Some studies conclude that DON can be transferred from dairy cows to their milk. Nevertheless, Keese *et al*. showed that there were no DON contamination of milk coming from dairy cows that were fed diets containing DON and other *Fusarium* toxins. However, the presence of its metabolite de-epoxy-DON in the quantity of 1–1.5 mg/kg was demonstrated (Keese *et al*., 2008). On the other hand, simulation of DON metabolism predicts the DON concentration in milk 1 mg/kg (Coffey & Cummins, 2008), but this simulation have not been confirmed experimentally

### Acute toxicity

Numerous DON toxicity studies in animals have targeted a specific toxicological outcome or mechanism, and thus provided insight into potential hazards (Pestka & Smolinski, 2005). DON is less toxic than other trichothecenes such as T-2 toxin, however, extremely high DON doses (*i.e.* unlikely to be encountered in food) can cause shock-like death. LD_50_ for mice ranges from 49 to 70 mg/kg (intraperitoneal DON injection) (Forsell *et al*., 1987) and 46 to 78 mg/kg (oral DON administration) (Yoshizawa *et al*., 1983). LD_50_ for 10-day old duckling is 27 mg/kg when the toxin is administered subcutaneously (Yoshizawa & Morooka, 1973) and 140 mg/kg for 1-day-old broiler chicks with DON oral administration (Huff *et al*., 1981; Pestka, 2007).

Male B6C3F1 mice were orally administered DON (25 mg/kg [84 µmol/kg]) to assess the kinetics of DON distribution and clearance. DON was detectable in the plasma, liver, spleen, and brain from 5 minutes up to 24 hours post administration. DON was detectable also in the heart and kidney from 5 minutes up to 8 hours post administration. The highest plasma concentrations were detected from 5 to 15 minutes after dosing. DON concentrations (mg/kg) in other tissues 5 minutes after dosing were 19.5 ± 1.9 in liver, 7.6 ± 0.5 in kidney, 7.3 ± 0.8 in spleen, 6.8 ± 0.9 in heart, and 0.8 ± 0.1 in the brain (Pestka *et al*., 2008). Clearance followed two-compartment kinetics (t(1/2)α=20.4 minutes, t(1/2)β=11.8 hours) (Pestka *et al*., 2008). The other study shows that in male B6C3F1 mice with orally given DON (25 mg/kg [84 µmol/kg]), the highest levels of DON were detected in kidney, heart, plasma, liver. thymus, spleen and brain 30 minutes post administration.

### Short-term and sub-chronic exposure

Overall, studies showed that short-term and sub-chronic exposure to DON decreased body weight, weight gain, and feed consumption in rats and mice. Haematological effects were also observed. Conflicting results are observed for the effect of DON on organ weights reported that spleen and liver weights and the liver-body and kidney-body weight ratios increased in Sprague-Dawley rats gavaged with DON (Pestka, 2007; Pestka & Smolinski, 2005). In the other studies, there is reported no effect on organ weight or organ-body weight ratios in rats and mice (Gouze *et al*., 2006; Sprando *et al*., 2005). DON induced lesions in the non-glandular stomach, and caused thymic lymphoid depletion, increased incidences and mean severity of spleenic haematopoiesis, and increased mean severity of sternal bone marrow adipocyte deposition in rats at the highest dose (Sprando *et al*., 2005).

### Chronic toxicity

Female B6C7F1 mice (7 weeks old) were fed experimental diets for 16 weeks that contained DON (20 mg/kg [67 µmol/kg]). DON reduced the mean daily food consumption (2.94 ± 0.66 g vs. 3.6 ± 0.48 g), the mean body weight gain (2.76 ± 0.84 g vs.12.94 ± 1.68 g), and total body weight, and increased serum immunoglobulin A (IgA) levels in treated vs. control mice starting 8 weeks after diet initiation. Serum IgA immune complex (IgA-IC) levels and mesangial IgA deposition increased starting 16 weeks after diet initiation. DON also increased *ex vivo* IgA secretion from the spleen and Peyer's patches (Iverson *et al*., 1995).

### Synergistic/antagonistic effects

In *Salmonella typhimurium* strain TA98, the combination of DON and aflatoxin B1 (AFB1) had a greater mutagenic effect than AFB1 alone. Additionally, a synergistic interaction between DON and nivalenol (NIV) was found (Tajima *et al*., 2002). The direct toxic effect of DON on the growth and on the expression of Salmonella pathogenicity island 1 (SPI-1) and SPI-2 virulence genes of *Salmonella Typhimurium* was also determined. At low non-cytotoxic concentrations, as it can be found in the serum of pigs, DON did not have any effect on either growth or virulence gene expression of *Salmonella Typhimurium*. However, higher concentration DON (0.025 g/mL) significantly promoted the uptake of *Salmonella Typhimurium* into macrophages. These results suggest that low but relevant concentrations of DON modulate the innate immune system and could thus increase the susceptibility of pigs to infections with *Salmonella Typhimurium* (Vandenbroucke *et al*., 2009).

A synergistic carcinogenic effect was observed in NIH mice when Sterigmatocystin and DON were both administered. The number of animals with lung adenocarcinomas and glandular stomach dysplasia increased. In mouse fibroblast L929 cells, a mixture containing DON, NIV, T-2 toxin, zearalenone, and fumonisin B1 produced greater inhibition of DNA synthesis than with treatment of each mycotoxin alone. Additionally, a synergistic interaction between DON and NIV has been determined (Madhyastha *et al*., 1994).

### Cytotoxicity

Numerous studies have been conducted in a variety of cell lines to assess the cytotoxic effects of DON. In mouse thymocytes *in vivo*, DON (0.5–8.0 mg/kg [2–27 µmol/kg]) dose-dependently induced significant increases in apoptosis rates compared to controls. In addition, DON (4 and 8 mg/kg [13–27 µmol/kg]) significantly decreased the proliferation indexes of the treated cells (Bony *et al*., 2007; Ouyang *et al*., 1996). Explants from weanling pigs were exposed to 0, 0.2, 1, 5 µM DON in the culture medium for 4 h. Preliminary cultures had shown that 10 and 30 µM DON induced necrosis of the explants after 4 h of incubation (Kolf-Clauw *et al*., 2009).

### Reproductive and teratological effects

In three-month-old nulliparous female NMRI mice, intraperitoneal injection of DON (3.3, 4.2, 5, or 10 mg/kg [11, 14, 17, or 34 µmol/kg] on gestation days 7 and 9 or 1.6, 2.5, or 3 mg/kg [5.4, 8.4. or 10 µmol/kg] daily on gestation days 7–10) produced high maternal deaths at the two higher doses. In embryos, the number of resorptions was dose-dependently increased in treated animals compared to controls. Skeletal abnormalities were observed. Exencephaly was mainly seen at 75 or 100 µg/30 g during the four-day treatment. At the higher dose and shorter exposure period, neural arch defects or fusion were mostly detected. In both experiments, vertebral bodies showed various deformities, hemivertebrae (except with 75 µg/30 g given for four days), and fused, branched, and/or cervical ribs (Debouck *et al*., 2001).

### Carcinogenicity

The International Agency for Research on Cancer (IARC) concluded in 1993 that “There is inadequate evidence in experimental animals for the carcinogenicity of deoxynivalenol.” Overall, DON was placed in Group 3, “not classifiable as to its carcinogenicity to humans.” (Some naturally occurring substances: Food items and constituents, heterocyclic aromatic amines and mycotoxins, www.iarc.fr). Subsequently, a two-year carcinogenicity study in mice was published. Dietary administration of DON did not result in an increased incidence of neoplasms in males or females. Particularly, in males, there was a decreased incidence of liver neoplasms, probably a result of lower body weights (Iverson *et al*., 1995).

### Genotoxicity

In Chinese hamster V79 cells, DON fractions from samples of wheat (30 ng/mL [0.10 µM]), barley (200 ng/mL [0.675 µM]), and corn (300 ng/mL [1.01 µM]) induced chromosome aberrations, mostly chromatid breaks (Hsia *et al*., 2004). DON (1–10 µmol) induced DNA damage in Vero cells (increased number of cells with long tails, tail DNA, tail length, and tail motion) in a dose- and time-dependent manner. Short-term incubations (4 hours) mainly induced an increase of the number of DNA fragments while longer incubation time (16 hours) mainly caused small size DNA fragments (Sun *et al*., 2002). Results from the Comet assay showed that DON increased mean tail moment in human Caco-2 cells in a dose-dependent manner at concentrations (0.01–0.05 µM [3–15 ng/mL]) that did not induce apoptosis. Furthermore, dividing cells exhibited greater sensitivity to DON than differentiated cells (Bony *et al*., 2007).

### Immunotoxicity

Many studies of host resistance, mitogen-induced lymphocyte proliferation, and humoral immune response have yielded a common theme that trichothecenes are both immunostimulatory and immunosuppressive depending on dose, exposure frequency and timing relative to functional immune assay (Pestka *et al*., 2004). Numerous immunotoxicity studies are cited in the JECFA monograph under the following topics: altered host resistance and humoral and cell-mediated responses, altered serum IgA levels, IgA-associated nephropathy, cytokine expression, and apoptosis in lymphoid tissue. Copious studies have since been conducted (Gray & Pestka, 2007; Chung *et al*., 2003; Kinser *et al*., 2005; Pestka & Zhou, 2000; Sugita-Konishi & Pestka, 2001; Uzarski *et al*., 2003; Wong *et al*., 2002; Yang *et al*., 2000). Additional *in vitro* studies showed that DON inhibited nuclear protein binding to NRE-A, an IL-2 promoter negative regulatory element, in murine lymphoma EL-4 T cells, induced cytotoxicity and apoptosis in WEHI-231 B cells, induced p38 activation, and increased IL-8 production (Zhou *et al*., 2005).

## Conclusions

Numerous investigations have been conducted in several animal species to clarify DON's tissue targets, mechanisms of action and limit doses for adverse effects. This approach provides a simple strategy that can be used to answer relevant questions of how dose, species, age, gender, genetic background and route/duration of exposure impact DON uptake and clearance in both animals and humans.
